# Understanding personal preferences to promote exercise adherence in Parkinson’s disease

**DOI:** 10.1016/j.prdoa.2025.100336

**Published:** 2025-04-28

**Authors:** Caro I. Cools, Sonja A. Kotz, Bastiaan R. Bloem, Nienke M. de Vries, Annelien A. Duits

**Affiliations:** a Faculty of Psychology and Neuroscience, Department of Neuropsychology & Psychopharmacology, Maastricht University, Maastricht, the Netherlands; b Radboud University Medical Centre; Donders Institute for Brain, Cognition and Behaviour, Department of Neurology, Centre of Expertise for Parkinson and Movement Disorders, Nijmegen, Netherlands; c Department of Human Movement Sciences, University Medical Center Groningen, University of Groningen, Groningen, the Netherlands; d Department of Psychiatry and Neuropsychology, Faculty of Health, Medicine and Life Sciences, School for Mental Health and Neuroscience, Maastricht University, Maastricht, the Netherlands; e Department of Medical Psychology, Maastricht University Medical Center, Maastricht, the Netherlands; f Department of Medical Psychology, Radboud University Medical Center, Nijmegen, the Netherlands

## Abstract

**Introduction:**

Exercise is a recognized treatment for people with Parkinson’s Disease (PwP), but personal exercise preferences are rarely considered in exercise intervention studies. Yet these are critical for understanding motivation and to ascertain long-term exercise adherence in PwP. This study compared preferred exercise types among PwP with exercise types investigated in prior research.

**Method:**

672 PwP participated in an online survey, answering questions about currently practiced and preferred exercise types. Besides, a brief review was performed, evaluating exercise types deployed in prior studies. The preferred exercise types of PwP were subsequently compared to exercise types used in prior research.

**Results:**

PwP mainly preferred the same exercises that they were currently practicing. These preferences did not necessarily align with exercise types reported in prior studies. The top 5 highly preferred sport categories but relatively little studied were walking, biking, swimming, boxing, and tennis.

**Conclusion:**

Future intervention studies may benefit from considering personal preferences to both strengthen and understand exercise adherence.

## Highlights

• PwP’s preferred exercise types align with their currently practiced exercise types.
• PwP’s preferred exercise types differ from exercise types used in prior studies.
• Walking, biking, swimming, boxing, and tennis are preferred but have been relatively little studied.
• Adherence to exercise is likely higher when considering personal preferences.

## 1 Introduction

Parkinson’s Disease (PD) is characterized by both motor and non-motor symptoms [1]. Exercising positively influences both. Maintaining physical activities may beneficially influence PD symptom progression [2], and exercising improves quality of life for people with PD (PwP) [3]. Moreover, one study suggested aerobic exercise to be associated with reduced global brain atrophy, enhanced functional connectivity in the brain, and improved cognitive control in PwP [4].

Despite potential benefits and guidelines about the type and degree of exercise to be performed [5], many PwP do not meet the recommended advice for physical activity [6]. One core factor stimulating exercise by PwP is motivation [7], which is associated with higher levels of premorbid motivation, greater self-compassion, and lower perceived disease severity [8]. Consequently, long-term exercise adherence might be stronger when exercise is linked to personal preferences in PwP [9].

Exercise intervention studies in PD often rely on a one-size-fits-all approach, and have not yet considered whether this approach aligns with exercise preferences in PwP. However, non-adherence to exercise might result from discrepancies between what PwP prefer and what intervention studies offer [10]. Although suggestions for personalized exercises were provided [11], little is currently known about how exercise preference might impact exercise adherence.

Therefore, exercise preferences in PwP in relation to practiced exercises were evaluated, and, second, these preferences were compared to exercise types investigated in prior exercise intervention studies. To answer the first objective, data were extracted from an online survey, and exercises PwP participated in (“practiced exercises”) were compared to those PwP actually preferred (“preferred exercises”). To answer the second objective, a brief literature review was performed. The observed exercise preferences of PwP were compared to exercise types in prior studies (“exercise types in prior studies”). Hypothesized was 1) no discrepancy between preferred and practiced exercises, and 2) a discrepancy between preferred and exercise types in prior studies.

## 2 Method

### 2.1 Preferences and practiced exercise types

Participants with a self-reported PD diagnosis were invited through multiple social channels to participate in an online survey. As one of many items in newsletters, it is unclear how many participants opened or read the newsletter, and proceeded to click on the survey invitation. Of those approached, 719 people responded. The online survey started with demographics, followed by questions about practiced and preferred exercises per exercise category. ‘Preferred’ refers to the willingness to participate in a specific exercise category, and participants could select an unlimited number of preferred exercise categories. The exercise categories were based on the LASA Physical Activity Questionnaire [12] and supplemented by interventions seen during the brief review. See Appendix A for the complete survey.

Based on the survey, both the percentages of practiced exercise types and preferences were calculated. As participants could select an unlimited number of preferred exercises, the number of preferred exercise categories could exceed 100 %.

### 2.2 Exercises types in prior studies

A brief review was conducted to identify exercise intervention studies for PwP published between January 2019 and June 2024. This timeframe was chosen to ensure an analysis of the most recent advancements in exercise intervention studies, mirroring the rise in such studies. The title and/ or abstract keyword methods of “Parkinson” AND “intervention” were used and 222 articles met the eligibility criteria. The remaining exercise interventions were divided into 30 different exercise types. Percentages were calculated per exercise type for the number of intervention studies performed. Next, the percentages of the exercise types in prior studies were compared with those of the preferred exercise types of PwP. A complete description of the methods section is provided in the supplementary material (see Supplementary Material).

## 3 Results

A total of 719 people responded; 54 participants were excluded from further analyses primarily because of missing data (see supplementary material). The final sample size was 665, including 92 incomplete surveys (completed more than the mandatory number of questions).

Participants in the final sample were on average 65.63 years (SD = 8.25, range: 38–88 years). 42.6 % were female, 57.1 % male, and 0.2 % identified as ‘other’ (and one missing value). Their mean self-reported health was 9.80 (SD = 3.86, min = 6, max = 25), with an average disease duration (time since diagnosis) of 6.66 years (SD = 5.06, range: 1–52 years). See supplementary material for additional information on assessments. The final sample consisted of exercising participants, with only 6.6 % of participants (n = 44) currently not exercising at all.

93.4 % of participants indicated to exercise regularly. Of these participants, 19.1 % participated in specific exercises for PD. Two weeks prior to the survey, the mean time of exercising or being physically active was 13.38 times (SD = 10.62, range: 0 – 85, e.g., like walking a dog multiple times a day or using a bicycle on a daily basis). The three most popular exercises were walking (including stepping and being on a treadmill), biking (including spinning, home trainers, and mountain bikes), and fitness/ gymnastics (including resistance training, cardio, workouts, body pump, and gymnastic exercises).

The remaining survey questions confirmed 37.1 % of participants exercising less after a PD diagnosis, 20 % maintaining the same level, and 42.9 % increasing exercising after diagnosis. Looking at differences pre- and post-diagnosis: biking decreased 19.2 %, swimming/ aquatic sports decreased 10.1 %, and the remaining categories decreased less than 10 %. Fitness/ gymnastics, however, increased by 9.7 %.

### 3.1 Practiced versus preferred preferences

The survey indicated that PwP participated mainly in walking, biking, and fitness/ gymnastics. Additionally, they also preferred swimming, dancing, and boxing. So, while PwP expressed preferences for some of the 30 different exercise categories in the survey, on average, they preferred the same top three exercises they had engaged in during the last two weeks before the survey (Table 1, column 2 and 3). Looking at preference levels for all exercise categories, only ‘walking’ and ‘fitness/ gymnastics’ scored lower in preference than the personal exercise engagement over the last two weeks.

**Table 1:** An overview of participation frequency, participation levels, and preference for exercise next to conducted research (%).

**Sports**	**Practiced exercises (incl. specially for PD) %**	**Preferred exercises %**	**Prior research %**
Walking (including treadmill)	77.0	54.0	8.8
Biking (including spinning, exercising on a home trainer or mountain bike)	44.9	51.6	6.5
Fitness/ gymnastics (including resistance training, cardio, workouts, body pump, rope jumping, and milon circuit)	37.3	28.1	26.9
Swimming/ aquatic sports	13.2	28.1	3.7
Dance total	5.7	25.9	14.0
Tango	1.1	3.9	1.9
Ballroom	0.6	4.2	0
Samba	0.3	4.7	1.4
Brazilian dance	0	2.4	1.4
Foxtrot	0.2	4.5	0
Modern/ Improvisation	3.8	6.2	6.5
Tai Chi	1.8	13.1	3.7
Qi gong	3.5	9.6	4.2
Mindfulness	5.7	13.1	3.2
Yoga	13.6	23.3	4.2
Karate	0	3.0	0.5
Boxing	17.6	26.6	2.8
Bowlen/ skittles/ jeu de boules	0.9	10.4	0
Tennis/ badminton/ table tennis/ padel/ squash	11.6	21.7	0.5
Jogging/ running/ fast walking	10.7	15.6	0
Rowing	2.9	9.6	0
Sailing	0.2	7.1	0
Billiards	1.8	6.3	0
Fishing	0.8	1.8	0
Football/ basketball/ netball/ hockey	2.4	7.5	0
Volleyball/ baseball	1.4	7.2	0
Skiing	0.9	6.5	0
Robot balance training	0	7.5	1.9
Nordic walking	6.3	14.9	0.9
Other sports	6.5^a^	3.2^b^	18.5^b^
None	6.6		

^a, b^ See supplemented material for an overview of ‘other sports’.

### 3.2 Preferred versus exercise types in prior studies

**Fig. 1:**
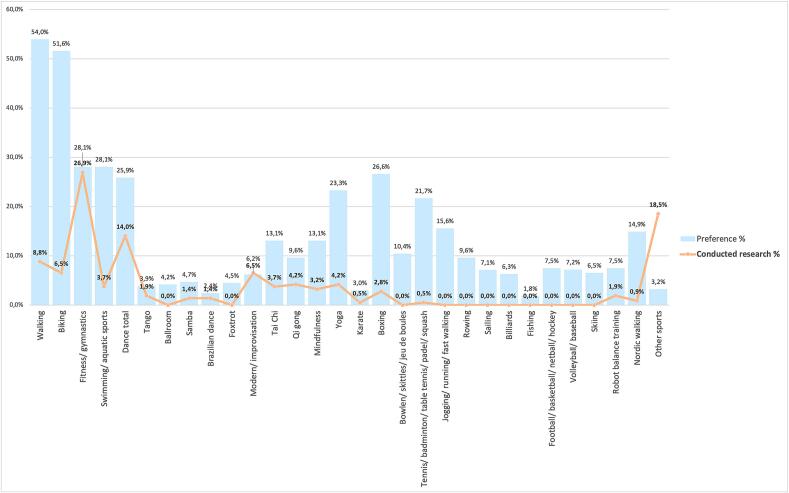
Overview of preferred exercise types (blue bars) and the exercise types in prior research (orange line). (For interpretation of the references to colour in this figure legend, the reader is referred to the web version of this article.)

Table 1 and Fig. 1 show distribution (in %) of exercise types in prior PD studies, and summarize the levels of participation and preferences for exercise types based on the online survey. PwP preferred walking, biking, fitness/ gymnastics, swimming, boxing, and dancing. However, the top 3 exercise types mostly used in prior research were fitness/ gymnastics, dancing, and walking. The table shows how 11 out of 30 exercise types were not included in any study since 2019.

Further, most exercise types showed a remarkable difference regarding preferred exercises and exercise types explored in prior research. The 10 exercise types showing the largest differences between preferred by PwP and used in prior studies were walking, biking, swimming/ aquatic sports, dancing, tai chi, yoga, boxing, jogging, tennis, and Nordic walking. These findings confirmed a discrepancy between preferred exercises and exercise types used in prior intervention studies.

## 4 Discussion

The present study highlights the need to consider exercise preference of PwP, to better understand exercise adherence. The survey clearly showed that practiced exercises mostly matched preferred exercises. Further, PwP reported preferences for walking, biking, fitness/ gymnastics, swimming, boxing, and dancing, whereas prior exercise intervention studies primarily investigated fitness/ gymnastics, and dancing. Such a discrepancy between preferred exercises and those investigated in prior studies may have resulted from an increased emphasis on those exercises that can lead to positive intervention outcomes. This might increase the likelihood that these exercise types rather than those preferred by PwP are likely to be chosen in future studies on exercise intervention [13].

Using a one-size-fits-all approach seems more feasible than studying a range of preferred exercises. However, the present results highlight the diversity and individual exercise preferences of PwP, supporting the need for personalized exercise interventions [14]. Personalized intervention is more attractive for PwP and can enhance enjoyment, happiness, and improve long-term and successful exercise adherence [9]. Looking at drop-out rates and the lack of considering personal preferences might explain why PwP do not respond well to selected exercise interventions. This needs to be further addressed in designs of future exercise intervention studies. This can already be achieved by asking participants about their preferences and whether the intended exercise interventions include their preferences.

The implementation of exercise interventions can only be successful with prolonged exercise adherence. Some questions in the survey displayed changes in type and exercise frequency pre- and post-diagnosis. These give insight into how some PwP remain motivated to exercise, whereas others are not. Future studies on exercise interventions should therefore include exercise preferences to better understand motivation and exercise adherence, and to further clarify why some PwP do (not) benefit from specific exercise interventions.

So far, it remains unclear to what extent PD symptoms, the options to exercise, or their motivation prevent PwP from practicing their preferred exercise. The survey did not allow to differentiate preferences due to physical abilities or severity in disease symptoms. Further light needs to also be shed on whether motivation to exercise in daily life is different from motivation to participate in intervention studies. Participating in exercise interventions that do not align with preferred exercises could be less attractive and undermine exercise adherence.

While the present results provide clear evidence that preferred exercises should be considered when designing new exercise intervention studies, some limitations were noted, such as a selection bias. PwP were only approached via social media, which might indicate that only proactive PwP responded. For example, the high percentage (93.4 %) of responders that actively exercise might not generalize to all PwP. Further, the online survey was conducted in Dutch, thus cannot generalize to PwP who speak other languages. Future studies should consider including an English version of the survey to broaden its applicability and comparisons between, at least, English- and other language speaking countries.

The self-reported exercise data could have also been biased. Participants may have interpreted ‘exercising’ differently, including daily activities such as 'walking' or 'biking’. This may have conflated how accurately one reports exercise frequency. Additionally, participants might have over- or underestimated their exercise activity levels. Because an online survey was used, it was not possible to control how consistently participants reported their exercise behavior. Future studies should expand the current type of research by performing interviews to obtain a better understanding of how exercise adherence could benefit from an objective activity tracking method to enhance response accuracy.

Importantly, the number of studies on a specific exercise type cannot reflect the quality of its effectiveness. The reported results can only indicate how frequently specific exercise interventions were studied. This study aimed at highlighting discrepancies between preferred exercises by PwP and those typically selected in exercise intervention studies.

Finally, this survey study limited the included brief review of exercise intervention studies in a relatively short time window. This choice was motivated by the exponential increase in specific types of exercise intervention studies during this time and allowed for a direct and immediate comparison of preferred and actually studied intervention exercises in PwP.

To conclude, exercise preference seems to be a missing but strong determinant in how successfully PwP adhere to exercise interventions. This study showed that PwP's preferred exercises align well with the exercises they practice, but not necessarily in PwP with exercises in prior intervention studies. To consider motivation and increase exercise adherence in PwP, personal exercise preferences seem to be a necessary factor to be considered in future exercise intervention studies.

## CRediT authorship contribution statement

**Caro I. Cools:** Writing – review & editing, Writing – original draft, Project administration, Methodology, Investigation, Formal analysis, Data curation, Conceptualization. **Sonja A. Kotz:** Writing – review & editing, Writing – original draft, Supervision, Conceptualization. **Bastiaan R. Bloem:** Writing – review & editing, Supervision. **Nienke M. de Vries:** Writing – review & editing, Supervision, Project administration, Methodology, Conceptualization. **Annelien A. Duits:** Writing – review & editing, Supervision, Conceptualization.

## Declaration of competing interest

The authors declare that they have no known competing financial interests or personal relationships that could have appeared to influence the work reported in this paper.

## Appendix A

Translated version (Dutch to English) of the online survey questions

1. General Questions (not derived from an official questionnaire)
1.1. What is your age?
1.2. What is your sex?
  • Male
  • Female
1.3. What is your nationality?
  • Dutch
  • Belgian
  • Other, ....
1.4. What is your marital status?
  • Married/ living together
  • Partnered, but not living together
  • Single
  • Widowed
1.5. What is your highest completed education?
  • Primary education
  • Lower vocational education (LBO, MAVO, VMBO)
  • HAVO
  • VWO (atheneum, gymnasium, lyceum)
  • Secondary vocational education (MBO)
  • Higher vocational education (HBO)
  • University (WO)
  • None of these (less than 6 years of primary education)
1.6. In which year were you diagnosed with Parkinson's disease?
1.7. In which year, looking back, did you first notice the initial symptoms?
1.8. Do you have symptoms on the left and/or right side?
  • Left side
  • Right side
  • Both left and right side
1.9. Do you have tremors?
  • Yes
  • No
1.10. Are you taking medication?
  • Yes
  • No
1.11. Do you have a dopamine/apomorphine pump?
  • Yes
  • No
1.12. Do you have a neurostimulator (DBS)?
  • Yes
  • No
1.13. Are you undergoing physiotherapy for Parkinson's disease?
  • Yes, individual sessions
  • Yes, through an exercise group
  • Yes, both individual sessions and an exercise group
  • No
1.14. Your balance is:
  • Good
  • Sometimes I need to grab onto something to avoid falling
  • I fall once a month
  • I fall once a week
  • I fall more than once a week
1.15. Your walking is:
  • Good, not affected
  • Affected, but I can still walk without aids
  • I need aids for walking
  • I can no longer walk
1.16. Do you feel like your feet are glued to the floor when you walk, turn, or when you start walking (freezing)? (derived from the FOG questionnaire)
  • Never
  • Very occasionally, about once a month
  • Sometimes, about once a week
  • Often, about once a day
  • Always when you walk
1.17. Have you had problems walking 1km? (derived from the PDQ-39 questionnaire)
  • Never
  • Occasionally
  • Sometimes
  • Often
  • Always
1.18. Have you had problems walking 100 meters? (derived from the PDQ-39 questionnaire)
  • Never
  • Occasionally
  • Sometimes
  • Often
  • Always
1.19. Do you have problems holding a glass or cup without spilling? (derived from the PDQ-39 questionnaire)
  • Never
  • Occasionally
  • Sometimes
  • Often
  • Always
2. Mapping current sport activities (based on work from another research group and adjusted by the brief review)

We would like to learn more about the sport(s) and activities you may have participated in recently. In the questions below, we will refer to this as “sport(s).” Through these questions, we aim to gain insight into how much you sport or have sported. Additionally, we would like to know which of these sports are specifically organized for people with Parkinson Disease and which are not.

2.1. Which sport(s) (excluding physiotherapy) have you practiced in the past two weeks that were not specifically organized for people with Parkinson's? (Multiple answers possible)
  • Walking
  • Cycling
  • Gymnastics (for seniors) / Sports and games for seniors
  • Swimming
  • (Folk) dancing
    – Tango
    – Ballroom
    – Samba
    – Brazilian dance
    – Foxtrot
    – Modern/improvisation
  • Tai chi
  • Qi gong
  • Mindfulness
  • Yoga
  • Karate
  • Boxing
  • Bowling / Kegeling / Jeu de boules
  • Tennis / Badminton / Table tennis
  • Jogging / Running / Speed walking
  • Rowing
  • Sailing
  • Billiards
  • Fishing
  • Football / Basketball / Korfball / Hockey
  • Volleyball / Baseball
  • Winter sports
  • Robot balance training
  • Nordic walking
  • Other sport, namely:
2.2. For each sport checked:
  • How many times have you practiced this sport in the past two weeks?_______times
  • How long do you usually spend on this sport each time?_______hours (number from 0 to 10)_______minutes (number from 0 to 59)
2.3. Are you currently participating in any sport(s) specifically organized for Parkinson's? Think of Dance for Parkinson's, Yoga4Parkinson, or Parkinson Boxing.
  • Yes
  • No
2.4. If so, which sport(s)? (Multiple answers possible)
  • Walking
  • Cycling
  • Gymnastics (for seniors) / Sports and games for seniors
  • Swimming
  • (Folk) dancing
    – Tango
    – Ballroom
    – Samba
    – Brazilian dance
    – Foxtrot
    – Modern/improvisation
  • Tai chi
  • Qi gong
  • Mindfulness
  • Yoga
  • Karate
  • Boxing
  • Bowling / Kegeling / Jeu de boules
  • Tennis / Badminton / Table tennis
  • Jogging / Running / Speed walking
  • Rowing
  • Sailing
  • Billiards
  • Fishing
  • Football / Basketball / Korfball / Hockey
  • Volleyball / Baseball
  • Winter sports
  • Robot balance training
  • Nordic walking
  • Other sport, namely:

3. Sport interests for future research

The following questions are about potential participation in future research on Parkinson Disease where you participate in sport(s) or activities for research.

3.1. Would you ever want to participate in a study for Parkinson's where you have to engage in sports?
  • Yes
  • No
3.2. Would you want to choose the type of sport you participate in during the research?
  • Yes
  • No, I am fine with being randomly assigned
3.3. Which sport(s) would you like to do?
  • Walking
  • Cycling
  • Gymnastics (for seniors) / Sports and games for seniors
  • Swimming
  • (Folk) dancing
    – Tango
    – Ballroom
    – Samba
    – Brazilian dance
    – Foxtrot
    – Modern/improvisation
  • Tai chi
  • Qi gong
  • Mindfulness
  • Yoga
  • Karate
  • Boxing
  • Bowling / Kegeling / Jeu de boules
  • Tennis / Badminton / Table tennis
  • Jogging / Running / Speed walking
  • Rowing
  • Sailing
  • Billiards
  • Fishing
  • Football / Basketball / Korfball / Hockey
  • Volleyball / Baseball
  • Winter sports
  • Robot balance training
  • Nordic walking
  • Other sport, namely:
3.4. For each chosen sport:
  a. Why are you interested in participating in this sport?
    • I have done this before in my personal life
    • I have done this before during a sport intervention for a study
    • Other people in my surroundings have done this sport before and were positive about it
    • I would like to try new things
    • Other, namely
  b. Does this intervention suit you?
    • Yes
    • No
  c. Are you motivated to participate in this intervention?
    • Yes
    • No
3.5. Do you think you would be more motivated if you could choose which type of sport to do?
  • Yes
  • No
