# Supplementary material for: Understanding personal preferences to promote exercise adherence in Parkinson’s disease
Source: Clin Park Relat Disord. 2025 Apr 28;12:100336. doi: 10.1016/j.prdoa.2025.100336 (PMC12133706; doi:10.1016/j.prdoa.2025.100336)
Supplement: Supplementary data 1 [file mmc1.docx]

**Supplementary Material**

**Method**

An online survey was used to collect the observational information on attendance, preference, and interests in different sport exercises of PwP. Furthermore, a brief but systematic search of the literature was used to collect relevant exercise intervention studies in PD. Both information sources were compared to pinpoint discrepancies and similarities in type of exercise interventions.

***Participants***

Various channels were used to invite people with PD (PwP) to the online survey. As this invitation was one of many items in newsletters from multiple platforms, one cannot conclude how many participants opened, read, and clicked on the survey invitation. PwP were included based on a self-reported diagnosis of PD, and excluded while reporting a form of atypical parkinsonism. There were no other in- or exclusion criteria. Of those approached, 719 people responded to the online survey. Participants could stop at any time without any further explanation.

***Procedure***

***Survey***

An online survey was created, in which PwP could participate anonymously in a period between November 2021 and March 2022. Various channels were used to recruit participants: a newsletter of ParkinsonNEXT (an online platform facilitating engagement in research for PwP and their peers), through readership of the Dutch Parkinson Society’s website and newsletters, as well as via the Parkinson Cafés (monthly gatherings for PwP and their peers) in several cities in The Netherlands (Maastricht, Sittard-Geleen, Valkenswaard, and Lelystad). The study was performed in accordance with the Declaration of Helsinki. This type of study does not require additional ethical approval as it is anonymous and does not contain any compromising information. The online survey took around 30-45 minutes, and participants could stop at any time without obligations.

The online survey started with demographics: including age, time of diagnoses, and self-reported health. Self-reported health was measured by evaluating balance (item 1.14.), walking in general (item 1.15.), freezing (item 1.16.), walking 1 kilometer (item 1.17.), walking 100 meters (item 1.18.), and holding a cup (item 1.19.). Together, these items resulted in a minimum and maximum score of 6 to 29, respectively. Higher scores were indicative of being more affected by PD symptoms.

Following, a list of exercises was given, accompanied by questions about previously practiced and preferred sport exercises. The categories of exercise interventions were based on the LASA Physical Activity Questionnaire [11] and adjusted to most common exercises as found in the brief review. While answering the survey, PwP could select a limitless number of sport exercises they were interested in. See Appendix A for the complete survey.

The goal was to collect information about participation in exercise types during the last two weeks, about participation in exercise types before diagnosis, and about preferences in future sport exercises. A comparison between participated and preferred exercise types was used to answer the first objective of the study, whereas the information regarding exercise preference was used in comparison to the brief review and to answer the second objective. Surveys missing questions about sport preferences were excluded from analyses.

A total of 719 people responded to the online survey. Subsequently, 54 participants were excluded from further analysis (n = 22 did not meet the diagnostic criteria for PD; n = 25 contained missing data regarding the questions about sport preferences; n = 7 had data entries containing values that were determined to be implausible and likely containing typographical errors. More specific: for times walking per two weeks numbers above 50 were excluded, reasoning people will not go for over four walks a day and the given answer must by a typo. For all other exercises it was assumed to not participate more than twice a day, therefore participants with numbers above 28 were excluded). The final sample size for analysis were 665 PwP, consisting of 573 participants who completed the survey in its entirety. 92 participants completed the survey partially, meaning they answered all the mandatory questions including responses related to sport preferences.

***Systematic literature search***

A brief, systematic search in PubMed was conducted to identify exercise intervention studies for PwP using title and/ or abstract keyword methods of “Parkinson” AND “intervention”, starting from January 2019 up to June 2024. Of the 2,273 articles identified, 222 met eligibility criteria, excluding animal research, systematic or scoping reviews, meta-analyses, study protocols, voice treatment, and physiotherapeutic interventions.

In the survey, participants reported engaging in activities such as horseback riding, zumba, pilates, and miniature golf or golf, which were not listed as predefined options in the questionnaire. These sport categories are marked as ‘other sport’-option in the table (Table 1). There were 40 intervention studies falling into the ‘other sport’-option in the systematic search, meaning they could not be matched with one of the 30 categories. Of those, 10 studies including (immersive) virtual reality, 10 about telehealth/ telerehabilitation/ mobile health, 4 including stepping as exercises, 3 about augmented reality/ exergaming, 3 about pilates, 1 about archery, 1 including both aquatic and land-based exercises, 1 about climbing, 1 about horse riding, 1 about motor imagery, 1 including multicomponent training, 1 being vestibular rehabilitation, 1 including video gaming, 1 about trampoline training, and 1 about Wii sports.

***Statistical analyses of the observational data***

Various percentages were calculated indicating practiced and preferred exercises as well as exercise used in prior interventions studies.
